# Supplementary material for: Transcriptional and cell type profiles of cortical brain regions showing ultradian cortisol rhythm dependent responses to emotional face stimulation
Source: Neurobiol Stress. 2023 Jan 4;22:100514. doi: 10.1016/j.ynstr.2023.100514 (PMC9842700; doi:10.1016/j.ynstr.2023.100514)

PheWeb 2019

Bar Graph | Table | Clustergram | Appyter

Click the bars to sort. Now sorted by combined score ranking.

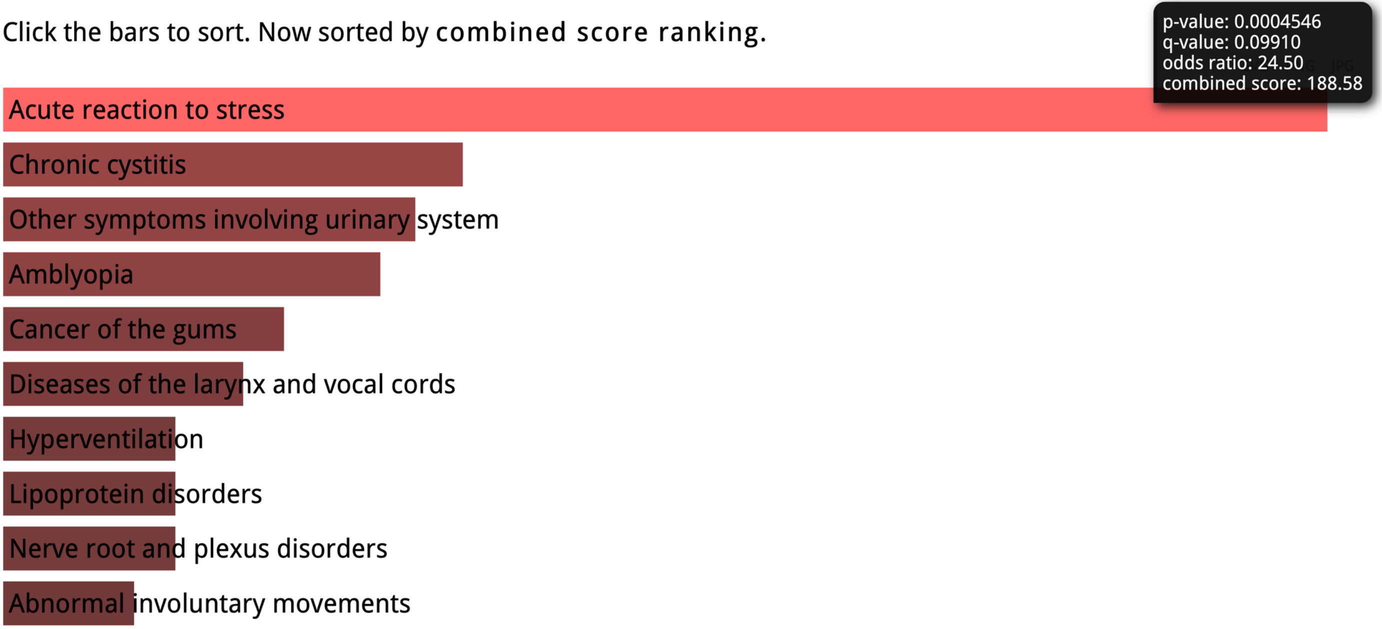

Supplement: Fig. S1 — Enrichment analysis results of the PheWeb database using the Enrichr web interface with as input the 223 differentially higher expressed genes from our fMRI-transcriptomics analysis. [file mmc1.pdf]
