# Supplementary figures and images for: Transcriptional and cell type profiles of cortical brain regions showing ultradian cortisol rhythm dependent responses to emotional face stimulation
Source: Neurobiol Stress. 2023 Jan 4;22:100514. doi: 10.1016/j.ynstr.2023.100514 (PMC9842700; doi:10.1016/j.ynstr.2023.100514)

Top20 tissues

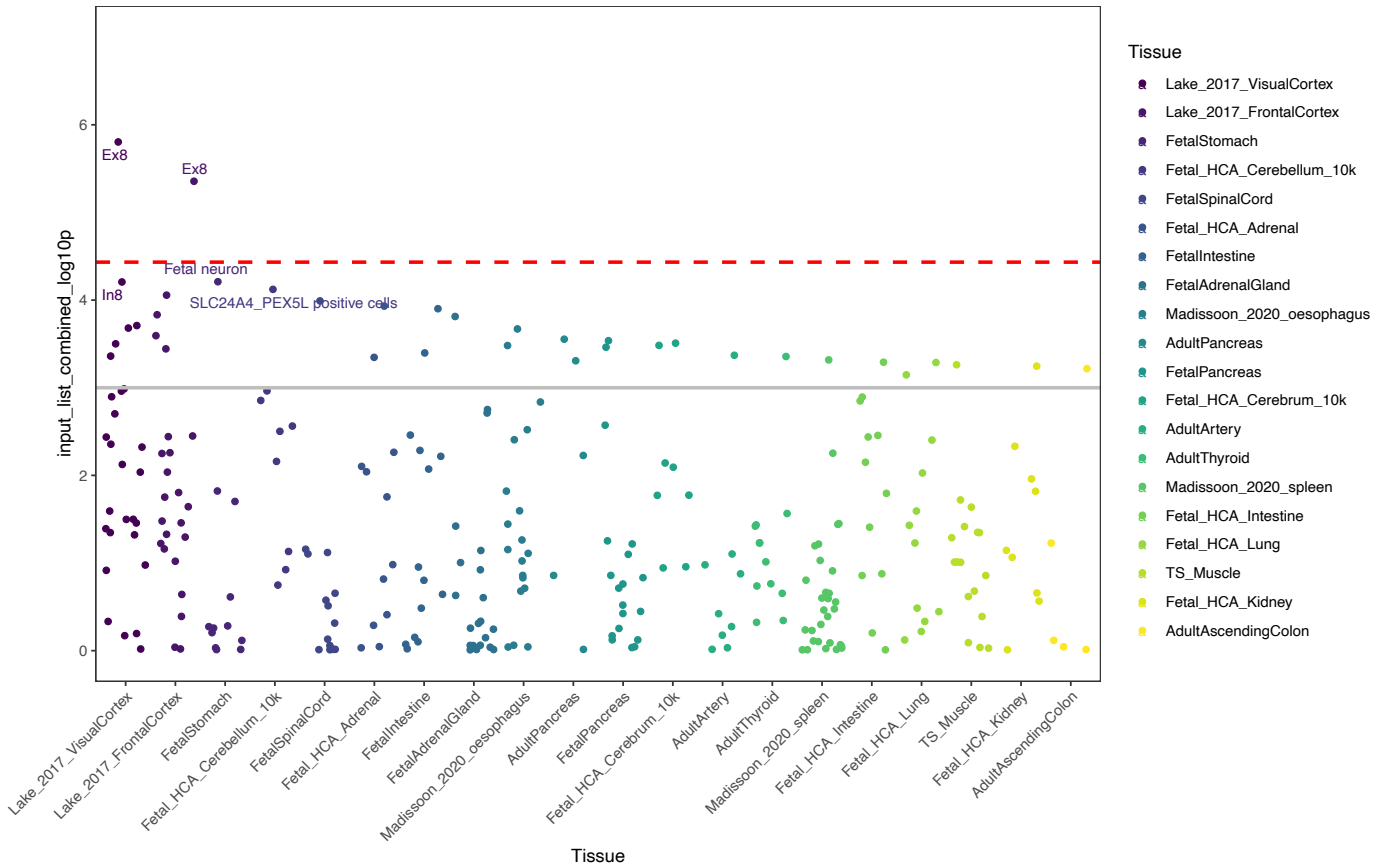

Top20 general cell types

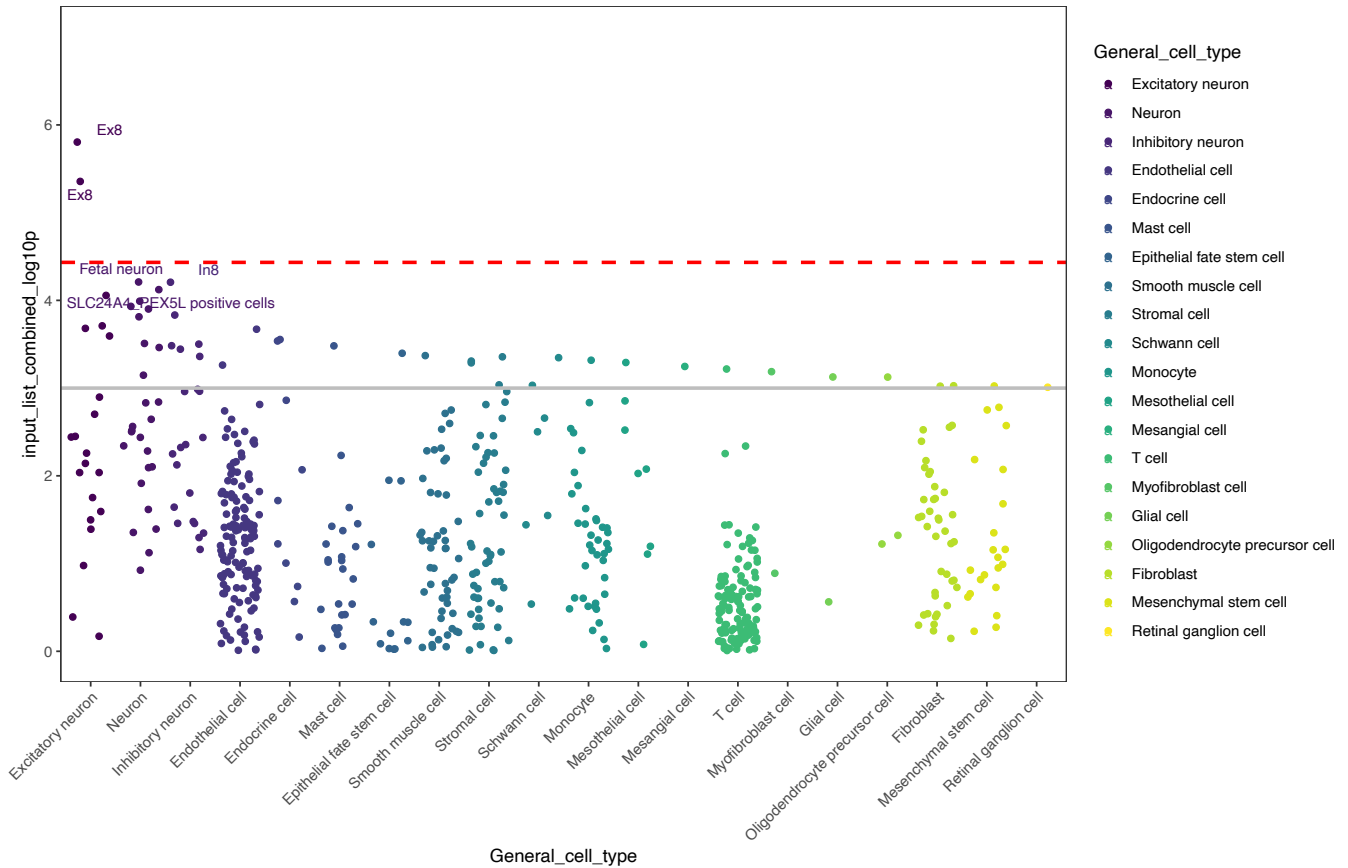

Supplement: Fig. S2 — WebCSEA results showing general tissue and cell type specificity, with the grey line indicating nominal significance (p = 0.001), and the red line indicating WebCSEA's default outputted threshold for significance (p = 3.69e-5). [file mmc2.pdf]

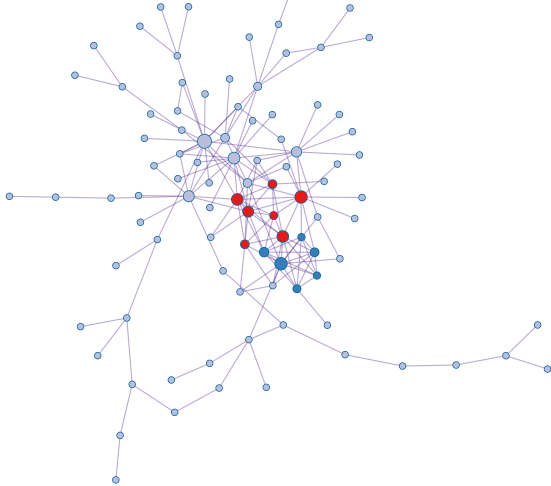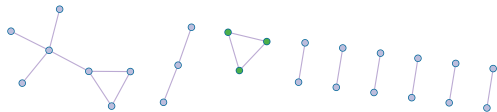

■ MCODE1

■ MCODE2

■ MCODE3

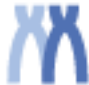

created by

<http://metascape.org>

Supplement: Fig. S3 — Metascape output graph of proteins coded by the differentially higher expressed genes and known physical interactions. Each node is a protein, and each edge represents a known physical interaction between the two proteins. The two densely connected subnetworks in the main graph are in red and blue. Proteins not included in the main graph are pictured on the right, with a separate densely connected network consisting of only three nodes pictured in green. There was no enrichment result for this isolated three-node network (and was therefore excluded from Fig. 4). [file mmc3.pdf]

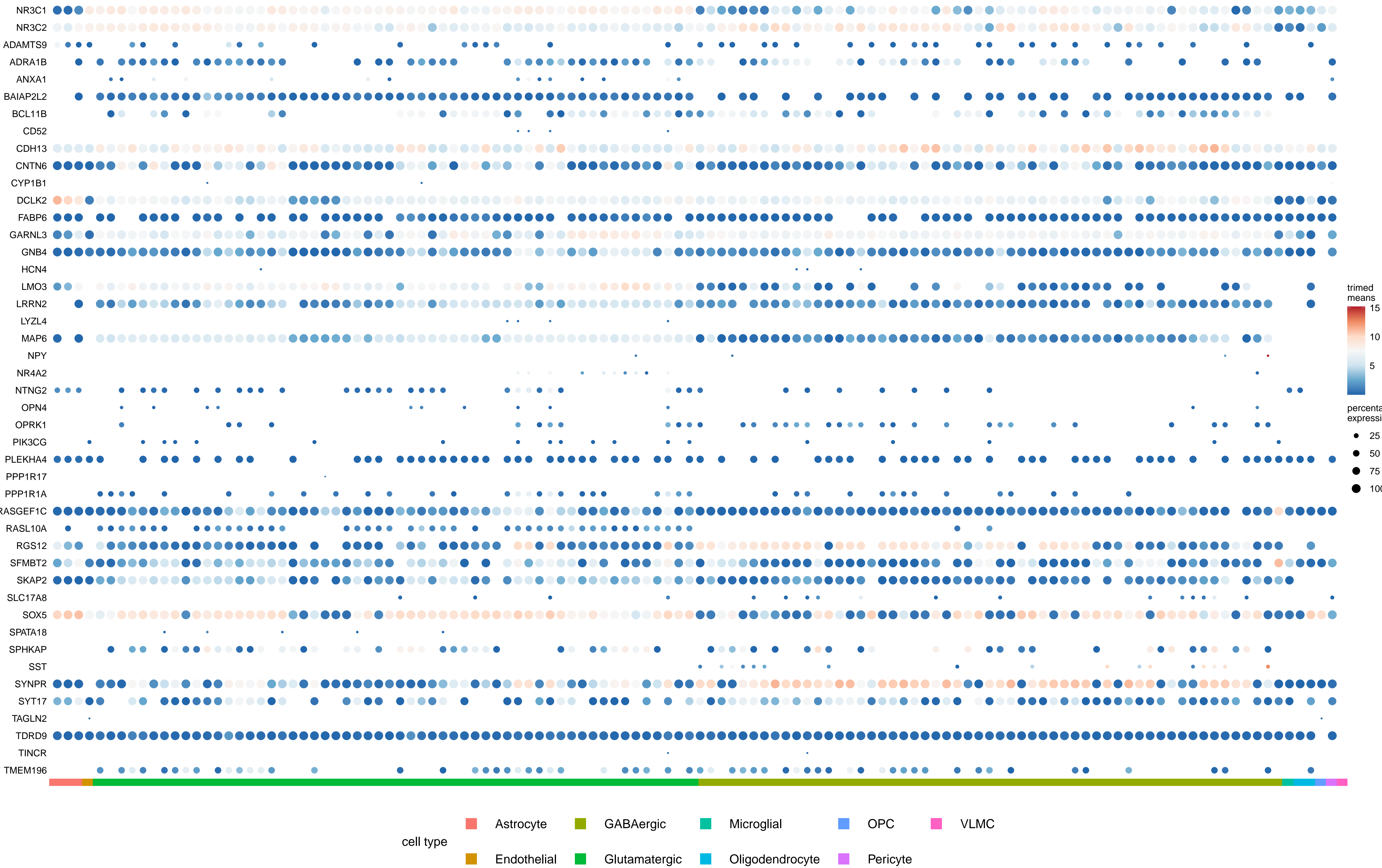

Supplement: Fig. S4 — Dot plot of trimmed mean gene expression (see Methods section for details) per cell type and their specificity (percentage of cell types expressing them). Genes included are GR, MR, and top-50 genes (higher differentially expressed). Genes with a trimmed mean expression of 0 in all cell types are omitted from the plot. [file mmc4.pdf]
